# Molecular Characterisation of Endogenous Vangl2/Vangl1 Heteromeric Protein Complexes

**DOI:** 10.1371/journal.pone.0046213

**Published:** 2012-09-28

**Authors:** Edwige Belotti, Tania M. Puvirajesinghe, Stéphane Audebert, Emilie Baudelet, Luc Camoin, Michel Pierres, Lea Lasvaux, Géraldine Ferracci, Mireille Montcouquiol, Jean-Paul Borg

**Affiliations:** 1 Inserm U1068, CRCM, Marseille, France; 2 CNRS UMR7258, CRCM, Marseille, France; 3 Institut Paoli-Calmettes, Marseille, France; 4 Aix-Marseille Université, Marseille, France; 5 Centre d′Immunologie de Marseille-Luminy, UMR 6102 INSERM/CNRS, Marseille, France; 6 Neurocentre Magendie, Planar Polarity and Plasticity Group, INSERM U862, Bordeaux Université, Bordeaux, France; 7 Aix-Marseille Université, CNRS, CRN2M UMR7286, Marseille, France; Baylor College of Medicine, United States of America

## Abstract

**Background:**

Mutations in the Planar Cell Polarity (PCP) core gene *Vangl2* cause the most severe neural tube defects (NTD) in mice and humans. Genetic studies show that the *Vangl2* gene genetically interacts with a close homologue *Vangl1*. How precisely Vangl2 and Vangl1 proteins interact and crosstalk has remained a difficult issue to address, with the main obstacle being the accurate discrimination of the two proteins, which share close sequence homology. Experimental evidence previously presented has been sparse and addressed with ectopically expressed proteins or with antibodies unable to biochemically discriminate Vangl1 from Vangl2, therefore giving rise to unclear results.

**Methodology and Main Findings:**

A highly specific monoclonal anti-Vangl2 antibody was generated and rigorously tested on both recombinant and extracted Vangl2 using surface plasmon resonance (SPR) analysis, western blot, and immunoprecipitation experiments. This antibody efficiently affinity-purified Vangl2 from cell lysates and allowed the unambiguous identification of endogenous Vangl2 by proteomic analysis. Vangl1 was also present in Vangl2 immunoprecipitates, establishing the first biochemical evidence for the existence of Vangl2/Vangl1 heterodimers at an endogenous level. Epitope-tagged Vangl2 and Vangl1 confirmed that both proteins interact and colocalize at the plasma membrane. The Vangl2 antibody is able to acutely assess differential expression levels of Vangl2 protein in culture cell lines, as corroborated with gene expression analysis. We characterised Vangl2 expression in the cochlea of homozygous and heterozygous *Lp* mutant mice bearing a point mutation within the C-terminal Vangl2 region that leads to profound PCP defects. Our antibody could detect much lower levels of Vangl2^Lp^ protein in mutant mice compared to the wild type mice.

**Conclusion:**

Our results provide an in-depth biochemical characterisation of the interaction observed between Vangl paralogues.

## Introduction

Planar cell polarity (PCP) is crucial for the regulation of tissue morphogenesis and embryonic development. For instance, PCP is involved in neural tube closure, in the orientation of hair bundle in inner ear sensory cells and of motile cilia in the embryonic node, as well as in asymmetric cell division [1]–[6]. A core set of signalling molecules referred to as core PCP proteins is required for PCP signaling and is highly conserved during evolution. Vangl/Strabismus is one of these core PCP genes and was originally identified in *Drosophila* where its function is mandatory for the correct development and function of eye, wing, and bristles [7], [8]. Mammalian orthologues of Vangl/Strabismus consist of two paralogues Vangl1 and Vangl2 [1], [9], [10]. In human, lethal missense mutations in *Vangl2* as well as in *Vangl1* have been identified in human embryos affected with severe neural tube defects [11], [12]. Mice carrying spontaneous mutant alleles of *Vangl2* such as the *Looptail* mutation (*Lp*) have clear PCP defects at homozygous state, including the most severe failure in neural tube closure (craniorachischisis), whereas *Lp* heterozygotes show only a mild phenotype [2], [13]. The *Lp* mutation also causes morphogenesis and patterning defects in numerous tissues, including stereociliary bundles misorientation in the cochlea [6]. Structurally, the *Vangl2* and *Vangl1* genes encode similar four-pass transmembrane cell surface proteins bearing intracellular cytoplasmic amino- and carboxy-terminal regions with two limited discontinuous extracellular loops [14]–[16].

If Vangl1 mutants do not display the severe NTD phenotypes observed in Vangl2 Lp mutants, it has been shown to genetically interact with Vangl2, and double heterozygous mice (*Vangl1^gt/+^/Vangl2^Lp/+^*) display a stronger phenotype than individual heterozygotes [17]. Notably, *Vangl1^gt^* ^/+^/*Vangl2^lp^* ^/+^ have a profound misorientation of outer hair cells (particularly OH3) stereociliary bundles whereas inner hair cells are unaffected [17]. In *zebrafish*, injection of *Vangl1* mRNA partially rescues the PCP defects of *Vangl2/trilobite* mutant embryos, suggesting that Vangl2 and Vangl1 have overlapping biochemical functions [10]. In the brain, comprehensive analysis of the pattern of expression of Vangl1 and Vangl2 shows both differences and overlaps [17], [18]. For example, Torban et al. ([16], [17]) showed that Vangl1 expression is restricted to the midline floor plate cells and to the notochord while Vangl2 is more widely distributed over the entire neuroepithelium and absent from the notochord [17], [18]. Later, and in areas of the midbrain, retina and telencephalon, Vangl2, but not Vangl1, is abundantly expressed [18]. In contrast, Vangl1 and Vangl2 are colocalized in the sensory epithelia of the mouse cochlea with a similar asymmetrical localization patterns in both hair cells and supporting cells (see Figure 4D and [19]). Altogether, these results show that in some systems, or at some developmental stages, Vangl1 and Vangl2 could interact in some specific protein complex. Vangl proteins have been hypothesized to act in a unique complex, yet no direct experimental proof has been provided to validate this hypothesis at the endogenous level. Studies continue to differ in their explanations of the Vangl2 *Lp* mutation. One body of evidence proposes that the mutation leads to a partial loss of function in a gene dosage-dependent pathway [15] whilst the other argues that it causes a negative dominance due to the alteration of the wild-type Vangl2 function by the mutant variant [19], [20]. Previous studies used immunohistochemistry or western blot to detect Vangl1 and Vangl2 in tissues of mutant animals without providing a complete characterisation of the sensitivity of antibodies, making interpretation of these results difficult [19].

In this study, we have generated a specific monoclonal anti-Vangl2 antibody able to robustly discriminate Vangl2 from Vangl1. This antibody was used in combination with proteomic analysis and resulted in the identification of an endogenous Vangl2/Vangl1 complex. Ectopic expression studies provided further evidence that Vangl2 and Vangl1 can form a unique protein complex at the plasma membrane and showed this interaction to be independent of the N- and C-terminal intracellular portions of Vangl2. The Vangl2-specific antibody was used to screen the differential expression of Vangl2 in culture cell lines and tissues. We provide data showing the high specificity of the antibody and confirmed that the *Lp* mutation severely impairs Vangl2 protein expression in murine cochlea. We provide convincing semi-quantitative *in vivo* biochemical evidence supporting the hypothesis that the *Lp* mutation alters the normal protein level of Vangl2. Taken together, our data shed light on the molecular mechanisms underlying the interaction between members of the Vangl protein family.

## Results

### Generation and characterisation of a highly specific anti-Vangl2 monoclonal antibody

Vangl2 and Vangl1 proteins belong to the Vangl protein family and are key members in mechanisms regulating PCP in vertebrates and invertebrates. The proteins have the same predicted tertiary structures and EMBOSS software highlights the extreme similarity of the proteins at the amino acid level, where human sequences have 64.3% amino acid identity and 78.6% similarity.

All antibodies directed against Vangl2 or Vangl1 are polyclonal antibodies, and are, in our hands and as reported [20], unable to accurately discriminate the two close human Vangl paralogues in immunoprecipitation experiments [16], [17].

**Figure 1:**
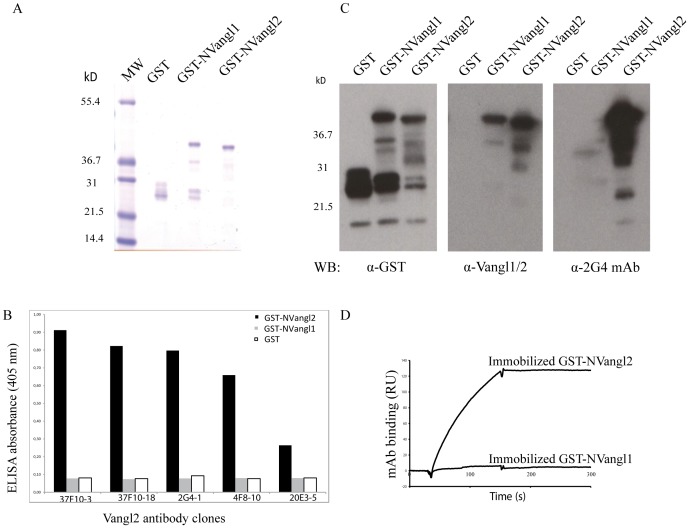
Generation of a highly specific anti-Vangl2 monoclonal antibody. (**A**) Expression of GST, GST-NVangl1 and GST-NVangl2 fusion proteins verified by SDS-PAGE and Coomassie staining. (**B**) Selected hybridomas were screened using an ELISA against GST, GST-NVangl1 and GST-NVangl2. mAb: monoclonal antibody. (**C**) Specificity of 2G4 mAb for GST-NVangl2 shown by western blot experiments, using antibodies specific for GST, Vangl2 and Vangl1/2. (**D**) SPR analysis using 2G4 mAb (10 µg/ml) injected over immobilized GST, GST-NVangl1 or GST-NVangl2. Sensorgrams show total signal from GST fusion proteins normalised with the non-specific signal (GST).

The N-terminal region is the most divergent region between the 2 proteins, therefore the region encompassing amino acids 1–102 was used for the generation of immunogenic sequences fused with a glutathione-*S*-transferase (GST) protein tag (GST-NVangl2). The corresponding region from Vangl1 was similarly fused to the GST as a control (GST-NVangl1). Both recombinant GST fusion proteins were produced in *E. coli* and were correctly expressed and purified as analysed by SDS-PAGE and Coomassie staining (Figure 1A). Monoclonal antibodies (mAbs) were raised against the GST-NVangl2 fusion protein in rat. A panel of these mAbs was able to discriminate the immunogen from GST and GST-NVangl1 in an Enzyme Linked ImmunoSorbent Assay (ELISA). A large number of antibody clones were tested in an ELISA assay and all raw data absorbance values (405 nm) for GST-NVangl2 are shown in Figure S1. A selection of highly and moderately positive clones is shown in Figure 1B, with corresponding data absorbance values of GST-NVangl1 and GST shown as controls. Among the positive clones, the 2G4 supernatant was indeed best able to specifically recognize GST-NVangl2, and so was further subcloned and chosen to be the optimal antibody candidate for subsequent experiments. When the 2G4 antibody was tested in western blot experiments for its reactivity toward 0.5 µg of GST proteins, we found that this antibody was only specific to GST-NVangl2 (Figure 1C). In this test, we clearly show the difference in specificity of the 2G4 antibody for Vangl2 over another widely used Vangl2 antibody (Vangl1/2) [21]. Surface plasmon resonance (SPR) analysis was next carried out by the immobilization of GST, GST-NVangl2 or GST-NVangl1 proteins onto surfaces derivatized with anti-GST antibody, and specific binding was assessed by flowing over purified 2G4 antibody. Only immobilized GST-NVangl2 was specifically recognized by 2G4 mAb (Figure 1D). In addition further SPR experiments were carried out by immobilizing the 2G4 mAb onto surfaces and flowing different concentrations of GST-NVangl2. Control experiments were repeated for GST-NVangl1 which did not show significant binding to 2G4 mAb even at high concentration of GST-Vangl1 (1 µM). Injection of various concentrations of GST-NVangl2 allowed the calculation of the constant of dissociation of 2G4 mAb with its antigen to be 0.41±0.16×10^−9^ M (Figure S2).

In order to further define the binding site of 2G4 mAb in the N-terminal part of Vangl2 used as immunogen (amino acids residues 1–102), a panel of different GST fusions proteins was produced in bacteria: GST-NVangl2 (1–110), GST-NVangl2 (1–80), GST-NVangl2 (1–60), GST-NVangl2 (1–40) and GST-NVangl2 (1–20). GST were purified on glutathione beads and loaded on a SDS-PAGE gel. All the proteins were recognized by 2G4 mAb by western blot, demonstrating that amino acids 1–20 of Vangl2 are recognized by 2G4 mAb. We provide a 2G4 mAb western blot showing that GST-NVangl2 (1–110) and GST-NVangl2 (1–20), but not GST alone, are recognized by 2G4 mAb (Figure S3). Comparison of the 20 first residues of Vangl1 and Vangl2 shows that the first 12 amino acids except one are identical between the two proteins. However, amino acids from 13–20 are quite different suggesting that the 2G4 epitope lies within this sequence.

### Identification of the endogenous Vangl1/Vangl2 protein complex

**Figure 2:**
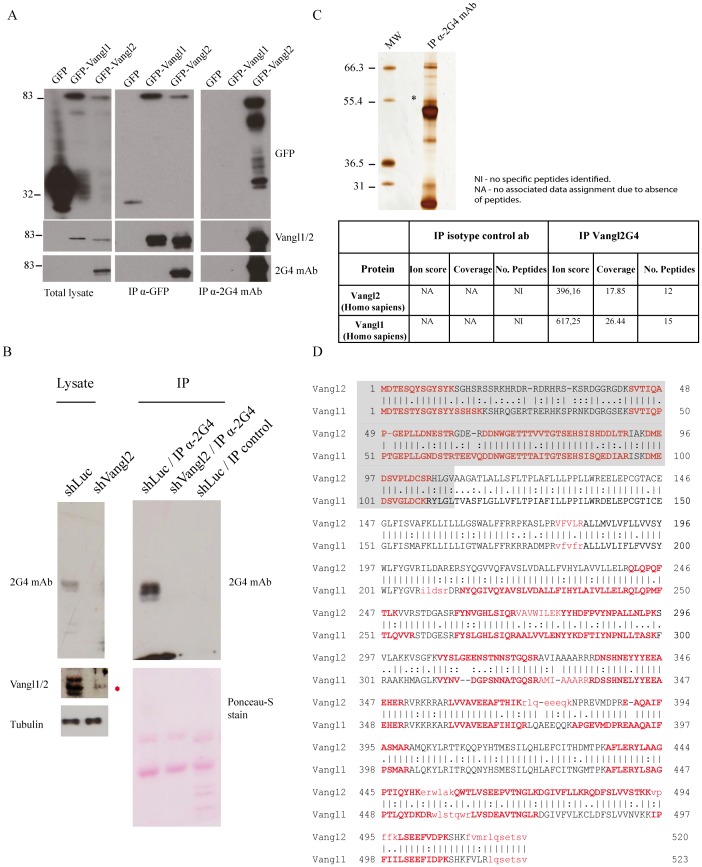
Identification of an endogenous protein complex containing the Vangl2 and Vangl1 proteins. (**A**) Specificity of 2G4 mAb was verified in western blot using T47D cell extracts expressing GFP, GFP-Vangl1, Vangl2 or GFP. Proteins were separated on SDS-PAGE and analyzed with western blot with specific antibodies. Specificity of 2G4 mAb in immunoprecipitation experiments GFP-Vangl1, GFP-Vangl2 or GFP expressed in T47D cells. Western blotting was carried out using 2G4 mAb, anti-GFP and Vangl1/2 antibodies. (**B**) SKBR7 cells were treated with shLuc or shVangl2 and assessed for Vangl expression. Vangl2 was immunoprecipitated from cell lysates and detected with the same 2G4 antibody. (**C**) Proteins were subsequently separated using Bis-Tris gradient gels and silver stained. Bands specific to 2G4 mAb were excised followed by in-gel trypsin digestion, chromatographic separation and orbitrap analysis. An asterisk indicates the bands corresponding to Vangl2. (**D**) Endogenous Vangl2 and Vangl1 were identified with the presence of multiple peptides and associated with a high probability Mascot scores (at least 40 arbitrary units). Bold characters indicate the peptides identified by mass spectrometry as shown with alignment of the two sequences. Regions shaded in grey correspond to the amino-terminal regions of Vangl1 and Vangl2.

The efficiency and specificity of 2G4 mAb to recognize full length Vangl2 was next confirmed in western blot experiments using GFP fusion proteins. Vangl1 and Vangl2 fused to an N-terminal GFP tag were expressed in T47D cells, a breast cancer cell line that does not express endogenous Vangl2 at the mRNA level (Figure S4). Proteins were extracted from cells expressing GFP, GFP-Vangl1 and GFP-Vangl2 proteins and consequently analysed by western blot. 2G4 mAb recognized GFP-Vangl2 but not GFP or GFP-Vangl1, even though all three proteins were readily detected by anti-GFP antibody (Figure 2A). As expected, the Vangl1/2 antibody recognized both GFP-Vangl1 and GFP-Vangl2 but not GFP alone. Therefore the 2G4 antibody is specific for Vangl2 in denaturing conditions.

The specificity of 2G4 mAb was also evaluated in immunoprecipitation experiments. Protein extracts from transfected T47D cells expressing GFP, GFP-Vangl1, or GFP-Vangl2 (Figure 2A) were subjected to immunoprecipitation with anti-GFP antibody or 2G4 mAb and precipitated proteins were revealed with anti-GFP, anti-Vangl1/2 or 2G4 antibodies (Figure 2A). Data clearly confirmed that GFP-Vangl1 and GFP-Vangl2 immunoprecipitated with anti-GFP antibody were recognized by Vangl1/2 antibody. 2G4 only recognized immunoprecipitated GFP-Vangl2. Furthermore, the 2G4 mAb was able to specifically immunoprecipitate GFP-Vangl2, but not GFP-Vangl1 or GFP, in non-denaturing experimental conditions (Figure 2A).

Next the 2G4 antibody was used as an affinity reagent to purify the endogenous protein from cell extracts. SKBR7 cells were chosen as they are expressing high levels of Vangl2 as assessed by RT-PCR and western blot (Figure S4). The monoclonal antibody was incubated with pre-cleared SKBR7 cell extracts treated with shLuc or shVangl2, and proteins eluted from the beads were separated by SDS-PAGE and analyzed using western blot experiments with the same antibody. Figure 2B showed that the 2G4 antibody was able to immunoprecipitate Vangl2, unlike the corresponding isotype-matched antibody control, in shLuc cell lysates and not shVangl2 cell lysates. Using Vangl1/2 antibody we detected Vangl1 in shVangl2 lysate (indicated with a red asterisk). Human Vangl2 is present as three major bands (around 60 kD) in SKBR7 lysates and was also evident in other cell lines, as detailed in the next section. A similar pattern with multiple bands has been shown to be generated by serine phosphorylation of the N-terminal region of Vangl2 [22]. In addition, immunoprecipitated proteins from SKRB7 cell lysates were migrated on a 16% SDS-PAGE (Figure S5). In these conditions, the band expression profile of Vangl2 was less resolved compared to the well-resolved band expression profile seen with the 7.5% SDS-PAGE of Figure 2B. Vangl2 appears as a single band at its predicted MW of 60 kD above the IgH. We blotted with anti-GADPH antibody and showed that GAPDH was not present in the immunoprecipitates and abundantly present in the lysate of SKBR7 cells.

The 2G4 monoclonal antibody was incubated with pre-cleared SKBR7 cell extracts and the proteins eluted from the beads were separated by SDS-PAGE followed by detection using a silver staining procedure (Figure 2C). The staining showed that only a selected number of proteins were immunoprecipitated by 2G4 mAb compared to the control (beads alone) (Figure 2C). Specific silver-stained bands from 2G4 mAb immunoprecipitation were selected and analysed using in-gel digestion, LC-separation and Orbitrap mass spectrometric analysis. Analysis showed that the band at approximately 60 kD contained endogenous Vangl2, which did not appear in the lane which used an isotype-matched antibody (HA antibody). Vangl2 was identified with good protein sequence coverage (17.85%) throughout the entire length of the protein (Figure 2C). Most of the identified peptides (highlighted in red characters) are associated with a high probability Mascot score (indicated by capital letters), and only a limited number of peptides are associated with a low probability Mascot score (indicated by simple letters) (Figure 2D). All identified peptides derived from Vangl2 are shown in Table 1. Closer inspection of the Vangl2 peptides showed the presence of a peptide with a high probability Mascot score and a reliable spectrum containing the first methionine with an additional N-terminal Arg-Ser-Asp-Ala sequence (peptide details are listed in Table 1 and supporting spectrum is displayed in Figure S6). The data provide further evidence for the existence of an additional sequence N-terminal to the conventional methionine of Vangl2 as suggested by others [23].

**Table 1:** List of peptides observed for Vangl2 identification.

Sequence	IonScore	Mascot Identity Threshold (MIT)	Mascot Homology Threshold (MHT)	Charge	m/z [Da]
SDAmDTESQYSGYSYK M4(Oxidation)	111	23	-	2	924.37311
SDAmDTESQYSGYSYK M4(Oxidation)	105	26	-	2	924.37183
VYSLGEENSTNNSTGQSR	101	33	-	2	971.94482
DNSHNEYYYEEAEHER	100	25	-	2	1042.91943
VYSLGEENSTNNSTGQSR	94	36	-	2	971.94452
SDAMDTESQYSGYSYK	83	24	-	2	916.37390
YLAAGPTIQYHK	72	37	24	2	681.36578
LSEEFVDPK	71	42	26	2	532.27094
SVTIQAPGEPLLDNESTR	63	36	-	2	963.99585
SVTIQAPGEPLLDNESTR	59	39	-	2	963.99518
FYNVGHLSIQR	58	37	24	2	667.35645
QWTLVSEEPVTNGLK	57	40	-	2	850.95111
DNSHNEYYYEEAEHER	57	28	-	3	695.61481
LSEEFVDPK	56	38	23	2	532.27081
FYNVGHLSIQR	56	40	25	2	667.35632
RQDFSLVVSTK	54	38	20	2	640.35620
QWTLVSEEPVTNGLK	53	37	17	2	850.95099
QDFSLVVSTK	53	36	23	2	562.30511
DNSHNEYYYEEAEHER	53	25	-	3	695.61414
EAAQAIFASmAR M10(Oxidation)	52	39	16	2	641.31873
DmEDSVPLDcSR-M2(Oxidation) C10(Carbamidomethyl)	52	29	-	2	720.29498
VYSLGEENSTNNSTGQSR	49	33	-	3	648.29846
RQDFSLVVSTK	49	35	20	2	640.35559
LVVAVEEAFTHIK	49	33	22	2	728.41620
DmEDSVPLDcSR-M2(Oxidation) C10(Carbamidomethyl)	49	26	-	2	720.29425
EAAQAIFASmAR M10(Oxidation)	49	36	16	2	641.31866
LSEEFVDPK	48	41	20	2	532.27075
YYHDFPVYNPALLNLPK	46	39	-	2	1032.53772
VYSLGEENSTNNSTGQSR	46	36	-	3	648.29840
EAAQAIFASMAR	45	40	16	2	633.32190
SVTIQAPGEPLLDNESTR	44	36	15	3	642.99872
EAAQAIFASMAR	44	37	15	2	633.32153
DMEDSVPLDcSR C10(Carbamidomethyl)	43	28	16	2	712.29785
SVTIQAPGEPLLDNESTR	42	39	19	3	642.99927

Mass spectrometry analyses also allowed the unambiguous identification of Vangl1 in the protein complex immunoprecipitated by 2G4 mAb (Figure 2C). Like Vangl2, Vangl1 was also identified with good protein sequence coverage (26.44%) (Figure 2C and 2D). All identified peptides derived from Vangl1 are shown in Table 2. The high specificity of the 2G4 mAb leads us to consider that Vangl1 was co-immunoprecipitated along with Vangl2 and that therefore these two paralogues can form a complex at the endogenous level.

**Table 2:** List of peptides observed for Vangl1 identification.

Sequence	IonScore	Mascot Identity Threshold (MIT)	Mascot Homology Threshold (MHT)	Charge	m/z [Da]
VYNVDGPSNNATGQSR	112	38	28	2	839.89563
VYNVDGPSNNATGQSR	108	35	28	2	839.89648
VYNVDGPSNNATGQSR	96	38	28	2	839.89581
IPFIILSEEFIDPK	96	35	-	2	830.96747
LVSDEAVTNGLR	80	38	23	2	637.34314
LVSDEAVTNGLR	80	40	23	2	637.34241
YLSAGPTLQYDKDR	76	40	28	2	813.91364
AALVVLENYYK	74	37	-	2	641.85815
DSSHNELYYEEAEHER	74	29	-	2	1004.42377
YLSAGPTLQYDKDR	71	37	24	2	813.91309
AALVVLENYYK	70	34	17	2	641.85809
DFTIYNPNLLTASK	66	39	22	2	798.91980
YLSAGPTLQYDK	65	37	-	2	678.34833
VYNVDGPSNNATGQSR	65	38	18	2	839.89685
VYNVDGPSNNATGQSR	64	35	22	2	839.89679
SVTIQPPTGEPLLGNDSTR	61	38	27	2	991.51770
DmEDSVGLDcK M2 (Oxidation) C10 (Carbamidomethyl)	54	28	-	2	642.76038
RDSSHNELYYEEAEHER	54	36	-	3	721.98560
AALVVLENYYK	54	38	-	2	641.85876
DMEDSVGLDcK C10(Carbamidomethyl)	53	31	17	2	634.76276
DFTIYNPNLLTASK	53	37	-	2	798.92157
SVTIQPPTGEPLLGNDSTR	53	35	22	2	991.51636

In order to further demonstrate specificity, we knocked down Vangl2 in cells endogenously expressing Vangl1 and Vangl2, and repeated the mass spectrometry analysis after purification with 2G4 mAb. If the 2G4 mAb is able to directly precipitate Vangl1, we would expect disappearance of Vangl2 and presence of Vangl1 in the immunoprecipitate of SKBR7 cells treated with shVangl2. We thus repeated immunoprecipitations using protein extracts from SKBR7 cells transfected with shLuc and shVangl2. We used SKBR7 lysates shown in Figure 2B. Note that Vangl1 is still present in shVangl2 treated cells (Figure 2B). Proteins were immunoprecipitated from SKBR7-shLuc or SKBR7-shVangl2 lysates with 2G4 mAb, run on a 7.5% SDS-PAGE, and stained with Imperial Blue Stain. The staining showed the presence of a 60 kD band above the IgH (asterisk) in shLuc but not in shVangl2 treated cell lysates that likely represents Vangl proteins. Bands at approximately 60 kD were excised in the two lanes (shLuc/IP 2G4 and shVangl2/IP 2G4 conditions) and extracted proteins were analysed by OrbiTRAP ESI-MS. In the shLuc/IP 2G4, we successfully identified Vangl2 and Vangl1, with a good number of peptides, all associated with good spectra and Mascot ion scores (see Figure S7). In addition all ion scores were above the Identity High values and cannot be considered as being artifacts. Neither Vangl2 nor Vangl1 peptides were found in the shVangl2/IP 2G4 lane. This provides strong evidence that the Vangl1 protein originally present in the IP reaction was brought by the heterodimerization with Vangl2 and not by the cross-reactivity of 2G4 mAb. In conclusion, these results clearly demonstrate that 2G4 mAb is a specific Vangl2 antibody.

### Characterisation of the Vangl1/Vangl2 protein complex using ectopic protein expression

**Figure 3:**
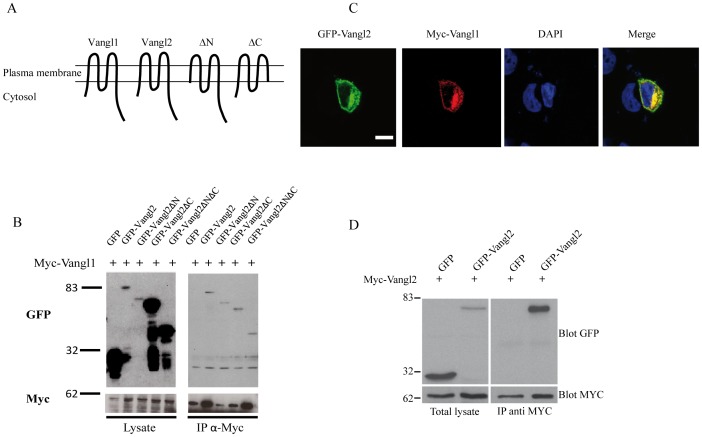
Interaction of epitope-tagged Vangl2 and Vangl1 proteins. (**A**) Schematic diagram of Vangl1 and full-length and truncated mutants of Vangl2 used for this study. (**B**) Transient co-expression of myc-Vangl1 with GFP-Vangl2, GFP-Vangl2ΔN, GFP-Vangl2ΔC, GFP-Vangl2ΔNΔC a control protein (GFP in T47D cells and immunoprecipitation with myc antibody shows co-immunoprecitation of Vangl2 with Vangl1). (**C**) Colocalization of ectopically expressed Myc-Vangl1 and GFP-Vangl2 in T47D cells and analysis by immunofluorescence and confocal analysis. All images were taken under a 40×objective. Scale bar corresponds to 10 µm and is labelled in white. (**D**) Vangl2 homodimerization is displayed by immunoprecipitation with Myc antibody shows co-immunoprecitation of Myc-Vangl2 with GFP-Vangl2. Protein complexes were separated using SDS-PAGE and western blot analysis.

In order to confirm the data obtained with endogenous proteins and evaluate how Vangl2 and Vangl1 interact, we next investigated whether a biochemical interaction existed between epitope-tagged proteins. Either myc-Vangl1 and GFP-Vangl2 or mutant GFP-Vangl2 (Figure 3A) constructs were expressed in the T47D cell line and co-immunoprecipitation experiments were performed (Figure 3B). Upon immunoprecipitation with anti-myc antibody, GFP-Vangl2, as well as GFP-Vangl2 devoid of the amino-terminal (GFP-Vangl2.ΔN) or carboxy-terminal (GFP-Vangl2.ΔC) regions or both the amino- and carboxy-terminal (GFP-Vangl2.ΔNΔC) regions, were all able to immunoprecipitate with myc-Vangl1. No interaction was found with a control GFP protein (Figure 3B). In addition it was shown that 2G4 antibody was able to immunoprecitate GFP-Vangl2 and consequently co-immunoprecipitate myc-Vangl1 (Figure S8). These results show that Vangl2 heterodimerizes with its paralogue Vangl1, and that the transmembrane regions and/or extracellular loops of Vangl2 are crucial for the interaction. Furthermore, in immunofluorescence experiments, GFP-Vangl2 and myc-Vangl1 proteins colocalize at the plasma membrane and in an uncharacterized perinuclear intracellular compartment of T47D cells (Figure 3C). Co-expression of Vangl2 fused to two different tags (GFP or myc) was also performed in T47D cells. Immunoprecipitation with anti-GFP antibody and western blot with anti-myc antibody showed that myc-Vangl2 was able to homodimerize with GFP-Vangl2 (Figure 3D). Therefore, Vangl2 has the capacity to form Vangl1/Vangl2 heterodimers as well as Vangl2/Vangl2 homodimers in living cells.

### Assessment of the differential expression of Vangl2 in culture cell lines and Looptail mutant mice

**Figure 4:**
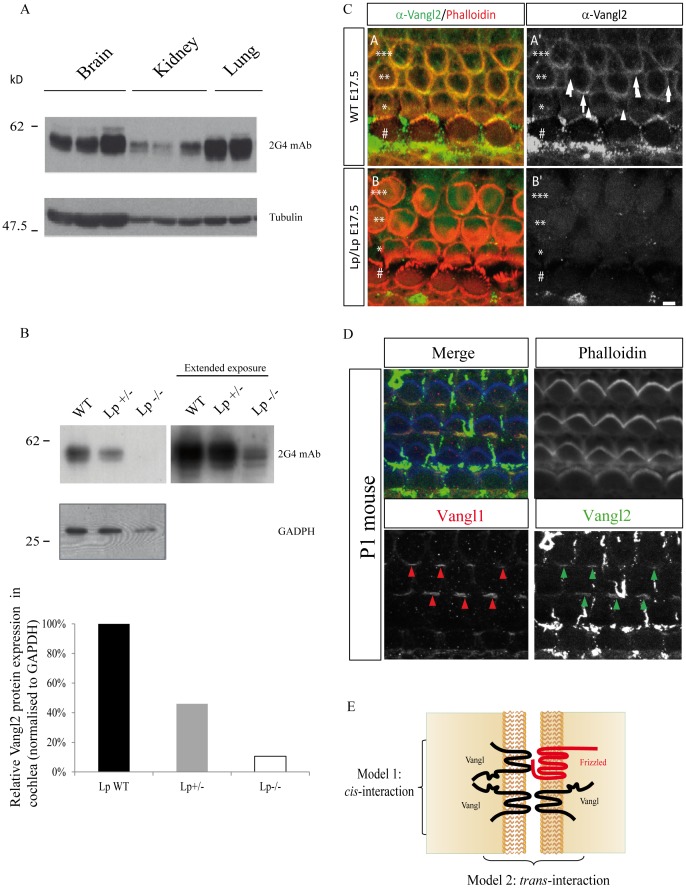
Differential expression of Vangl2 in different murine tissues and mouse cochlea. (**A**) Expression profile of Vangl2 from murine tissues (brain, kidney and lung) detected with 2G4 antibody in western blot. (**B**) Vangl2 protein expression in mouse cochlear tissue deriving from homozygous *Lp* and heterozygous Vangl2^Lp/+^ heterozygous mice compared to wild-type littermates, using 2G4 mAb antibody. Corresponding densitometry measurements representing relative Vangl2 protein expression normalized to GADPH protein levels. (**C**) Absence of staining with 2G4 mAb in Vangl2 mutant. Surface views of cochleae from WT (A-A’) and Looptail homozygote (B-B’) from E17.5 mice processed for immunocytochemistry with 2G4 mAb. At this stage, the cochlea comprises a single row of inner hair cells (#) and three rows of outer Hair Cells (OHC1 *, OHC2 **, OHC3 ***) surrounded by supporting cells. Phalloidin staining (actin, red) reveals hair cells borders. The image is taken at the level of the zonula adherens for the second and third row of OHC2 and OHC3, where Vangl2 accumulates. In the WT sample, we observe accumulation of Vangl2 at the junction between hair cells and supporting cells (A-A’, arrows). In contrast, in Lp/Lp cochlea, there is a complete absence of Vangl2 staining at the membrane of the cells (B,B’). Scale bar = 3 µm. (**D**) Co-localization of staining with 2G4 mAb and Vangl1 Ab. Surface view of a cochlea from a newborn mouse processed for immunocytochemistry with 2G4 mAb and Vangl1 Ab. The two antibodies reveal a co-localization of Vangl1 (A”) and Vangl2 (A’”) proteins at the junction between hair cells and supporting cells (arrows). Phalloidin is in blue, Vangl1 in red, and Vangl2 in green. Scale bar = 3 µm. Note: in the green channel, remains of the tectorial membrane covering normally the cochlear epithelium lead spots of non-specific green labeling. (**E**) Schematic diagram representing the two possible models of Vangl1/Vangl2 interaction.

We have chosen to investigate the differential expression of *Vangl* genes in cultured cell models, and screened human breast cancer cell lines as models. The mRNA expression level of *Vangl2* and *Vangl1* genes was analysed using semi-quantitative RT-PCR. As shown in Figure S4, *Vangl1* mRNA has a broad expression in all tested breast cancer cell lines whereas Vangl2 mRNA is more selectively expressed and only detectable in a limited number of cell lines. The mRNA expression of *Vangl1* and *Vangl2* in human cell lines mirrors that seen in developing murine tissues, as these genes have overlapping as well as non-overlapping patterns of expression in the developing neural tube [18], [24]. Protein extracts of breast cancer cell lines either expressing only *Vangl1* (T47D, MDA-MB-231) or expressing concomitantly *Vangl1* and *Vangl2* (SKBR7, SUM149), were evaluated for Vangl2 expression by western blots probed with 2G4 mAb. As shown in Figure S4, Vangl2 was only detectable in SKBR7 and SUM149 cells, which correlates exactly with *Vangl2* mRNA expression. The rabbit polyclonal Vangl1/2 was not able to discriminate Vangl1 from Vangl2 and showed uniform expression in all cell types regardless the Vangl2 status (Figure S4). We also used 2G4 mAb to evaluate expression of murine Vangl2 in brain, kidney and lung tissues from three (brain, kidney) or two (lung) different animals. These data clearly show that Vangl2 has a multiple band expression profile in murine tissues as seen with human cell lysates. This is more clearly seen in organs which have a higher expression of Vangl2 than brain and lung. However the complex band expression profile of Vangl2 is also evident in kidney which expresses comparatively lower levels of Vangl2 than in brain and lung (Figure 4A). The nature of bands in SUM149 cells recognized by the 2G4 mAb in western blot was further demonstrated to be specific for Vangl2 as knockdown using short hairpin RNA Vangl2 (shVangl2) leads to a near complete disappearance of the Vangl2 signal as compared with control short hairpin RNA (shLuc) (Supplementary Figure S9, panel A). There is also a near-complete disappearance of Vangl2 protein when SKBR7 cells are treated with 2 different siRNAs against human Vangl2 (Figure S9, panel B).

Given the sensitivity and specificity of the 2G4 antibody in assessing Vangl2 protein expression, the antibody was then used to characterize Vangl2 in homozygous and heterozygous *Lp* mutant mice compared to wild-type littermates. The S464N mutation within the C-terminal region of Vangl2 in *Lp* mouse mutants leads to severe PCP defects at homozygous state. This mutation decreases the stability of Vangl2 and its capacity to reach the plasma membrane [25], [26]. We analysed the expression of Vangl2 in the cochleae from wild-type (WT), Vangl2*^Lp/+^* or Vangl2*^Lp/Lp^* mice by western blot using the 2G4 mAb. As expected the analysis of Vangl2 expression in the cochleae of Vangl2*^Lp/+^* mice was much reduced as compared with Vangl2 expression in wild-type mice (Figure 4B). This decrease was even more pronounced in homozygous Vangl2*^Lp/Lp^* mice (Figure 4B, left panels). These data confirms *in vivo* that the S464N mutation dramatically compromises expression of the Vangl2 protein [24]. However, a longer exposure of the film showed that some Vangl2 protein remained in Vangl2*^Lp/Lp^* cochlea although at a much reduced level than in wild-type mice (Figure 4B, right panel). Quantitation of the relative protein expression of Vangl2 normalised to GADPH control protein levels showed a 54% decrease in Vangl2*^Lp/+^* compared to WT mice, whilst in Vangl2*^Lp/Lp^* 11% total Vangl2 remained in the cochlear tissue (Figure 4B). Taken together, our data show that the 2G4 mAb can reliably discriminate Vangl2 from Vangl1 in human and mouse protein extracts, and that homozygous *Lp* mutant mice express the mutant Vangl2 protein at much lower levels than wild-type mice. In order to validate expression of Vangl2 in tissues, immunofluorescence staining was carried out in the mouse cochlea (Figure 4C). Staining with 2G4 mAb (green) was strongly decreased in Vangl2*^Lp/Lp^* compared to Vangl2*^+/+^* confirming data obtained in western blot experiments (Figure 4B). We also performed co-staining using 2G4 mAb (green arrows) and Vangl1 antibody (red arrows) and demonstrated an overlapping distribution of the two proteins at the cell membrane of the stereociliary hair bundles (Figure 4D). This confirms previous data showing that Vangl1 and Vangl2 colocalize in the sensory epithelia of the mouse cochlea with a similar asymmetrical localization patterns at the junction between hair cells and supporting cells [19].

## Discussion and Future Directions

Until now, genetic evidence has been the strongest argument to support a relationship between members of the Vangl family of proteins. Precedent published work has documented the genetic interaction of Vangl2 with Vangl1, a close paralogue, in mice. However, this has only been correlated with limited biochemical experimental data, which analyzed solely ectopic protein expression. There has been a complete absence of biochemical experimental data studying the endogenous protein-protein interaction between Vangl2 and Vangl1. Expression of the endogenous Vangl proteins is complex in the developing embryo. During early neural development, Vangl2 was shown to be abundantly expressed in different parts of the brain, whereas Vangl1 was somewhat undetectable/absent [18]. Some overlapping expression of Vangl2 and Vangl1 exists in neuroepithelial cells of the floor plate. This colocalization decreases in the mature neural tube as Vangl1 expression is restricted to the midline floor plate region, whereas Vangl2 has a broader expression in the entire neuroepithelium [17], [18]. In this study we tackled the problem of the lack of endogenous biochemical data analyzing the interaction between Vangl proteins. Torban et al. characterized Vangl1 and Vangl2 specific antibodies in immunofluorescence assays and showed lack of specific staining in tissues deficient in Vangl species [16], [17]. In their published work, these polyclonal antibodies were not able to discriminate the proteins by immunoprecipitation. Due to a lack of availability of Vangl2 specific antibodies, we decided to generate a highly-specific Vangl2 antibody able to identify Vangl2 in tissues and cultured cells lines with high sensitivity. This antibody allowed the affinity purification of Vangl2 and the co-immunoprecipitation of one of its endogenous binding partner, Vangl1. This provided the first detection and comprehensive biochemical characterization of the endogenous forms of these PCP proteins, demonstrating the ability of Vangl2 and Vangl1 to physically associate and form a protein complex. We showed differential expression of the 2 Vangl paralogues in breast cancer cell lines which express either both Vangl paralogues or only Vangl1. We confirmed colocalization of the two Vangl paralogues by immunofluorescence in the mouse cochlea. Additionally, we found that Vangl2 can homodimerize in cultured cells. Therefore, Vangl proteins are able to homo- or heterodimerize, creating different Vangl complexes that may vary during embryonic development. It is currently impossible to quantify the ratio of Vangl1/Vangl2 heterodimers versus Vangl2/Vangl2 homodimers in cell lines and tissues. From our results, we believe that the transmembrane and/or extracellular loops of Vangl2 are implicated in its oligomerization (Figure 3B). The close proximity of the hydrophophic amino acid residues of the transmembrane domains may be responsible for the interaction between Vangl proteins within the lipid bilayer of membranes. Exposure of the two putative extracellular loops to the extracellular space has been suggested using insertion of epitope tag within the loops [14], [15] and biotinylation experiments [27]. Furthermore, studies in *Drosophila* support the ability of Vangl/Strabismus to interact in *trans* with the extracellular domain of Frizzled receptors allowing Frizzled to act non-autonomously [28]. This leaves open two possible modes of interaction between Vangl1 and Vangl2, i.e. a *trans* and a *cis* model as depicted in Figure 4E. Other proteins able to use to their membrane microdomains to form homotypic and heterotypic interactions are the small membrane proteins of the tetraspanin superfamily. These proteins are able to laterally cluster in order to facilitate cell signalling functions in cell adhesion and growth factor as well as co stimulatory proteins. They also have functions in antigen presentation because of interactions with major histocompatibility complex (MHC) I and MHCII molecules [29]. Our data suggest that the transmembrane domains of Vangl2 are sufficient for interaction. These data could be the basis of future investigations aiming to decipher more carefully the mode of interaction of Vangl proteins.

Taken together, this work sheds light on some molecular aspects of the genetic interaction between Vangl2 and Vangl1 identified in mouse models. Indeed, homozygous Vangl1 (Vangl1^gt/gt^) and heterozygous Vangl2^Lp/+^ mutant mice do not present strong PCP defects, in contrast to homozygous Vangl2^Lp/Lp^ and to double heterozygous Vangl1^gt/+^/Vangl2^Lp/+^ mice as well as Vangl1^gt/gt^/Vangl2^Lp/+^ mice [17]. Absence of strong phenotype in homozygous Vangl1^gt/gt^ mice highlights the predominance of Vangl2 in the Vangl family. Vangl2 can probably compensate Vangl1 loss through the formation of functional Vangl2 homodimers. However, it remains unclear how the combinatory loss of a single allele of Vangl1 and Vangl2 can lead to a strong PCP phenotype. One possibility, which has already been supported by recently published work [19], [20], could be that the Vangl2^Lp^ acts as a dominant negative protein by interacting with wild-type Vangl1 and Vangl2 proteins in double heterozygous Vangl1^gt/+^/Vangl2^Lp/+^ mice. Immunohistochemistry of the developing neural tube of *Lp* mutant embryos show that the Vangl2 protein expression is severely reduced and absent from the plasma membrane [24]. Recent *in vivo* data also show a redistribution of Vangl1 in the *Lp* mutant [20]. From the present work, some Vangl2 protein is still detected in homozygous *Lp* mutant cochlea and may act as a dominant negative protein by interacting with Vangl1. Indeed, we demonstrate here that the carboxy-terminal region of Vangl2, where the *Lp* mutation is located, is not required for the formation of Vangl1/Vangl2 heteromeric complexes (Figure 3B). Accordingly, epitope tagged Vangl2^Lp^ can still co-immunoprecipitate with epitope tagged Vangl1 [20]. As the Vangl2 *Lp* mutant is unable to reach the plasma membrane [25], [26], the Vangl2^Lp^/Vangl1 heteromeric complexes could be retained in intracellular compartments thus precluding proper signalling functions. Furthermore, in contrast to wild-type Vangl2, Vangl2^Lp^ is unable to bind Dishevelled. Therefore, the Vangl1/Vangl2^Lp^ complexes may be deficient for Dishevelled signalling. In summary, the *Lp* mutation provokes a range of defects affecting directly (stability, trafficking) or indirectly (formation of incompetent signalling heterodimers) Vangl1 and Vangl2 functions. Finally, overexpression of Vangl2 in vertebrates leads to PCP defects similar to Vangl2 loss-of-function mutants [3] (M.M., unpublished results). This overexpression may alter the stoichiometry of Vangl1-Vangl2 heterodimers favouring Vangl2 homodimers with potential deregulated functions. Another explanation is that overexpression may target Vangl2 to inappropriate sites and may in-turn lead to aberrant signalling in cells and tissues. Further studies will have to be carried out to gain an in-depth insight into the intricate complexity of Vangl2 and Vangl1 interactions and functions during embryonic development.

## Experimental Procedures

### Cell Lines, Cell Culture and Cell Transfection

SKBR7 (NCBI Gene Expression Omnibus: GSM75171 record, epithelial Breast tumor cell line SKBR7_b39_s31 [Homo sapiens]) cells were grown in RPMI medium supplemented with 100 U/mL of penicillin, 100 µg/ml of streptomycin and 10% heat-inactivated FBS. T47D cells (ATCC HTB-133, USA) were grown in accordance with ATCC recommendations. All cell lines were grown at 37°C in 5% CO_2_ incubator. T47D cells were transfected with pEGFP (GFP-Vangl2 or ΔN and ΔC mutants) using Lipofectamine 2000 reagent used according to the manufacturer's instructions (Invitrogen).

### Mouse husbandry and genotyping

Loop-tail wild-type, heterozygous and homozygous mice were all previously described in details [21]. All animal care related to this study was specifically approved and carried out in full accordance with the European Communities Council Directives (86/609/EEC) and the French national Committee (87/848) recommendations.

### Plasmids and Constructs

Murine normal length Vangl2 cDNA fragments were amplified by PCR from the Vangl2 cDNA. The PCR products were cloned using the Gateway™ technology (Invitrogen, France). SiRNA against human Vangl2 and non-targeting siRNA (control siRNA) were purchased from Dharmacon.

### RNA isolation, cDNA synthesis and PCR

Total RNA was isolated according to the manufacturer's protocol (RNeasy Mini kit, Qiagen), and RT-PCR was performed by using the SuperScript RT-PCR kit (Invitrogen) and separated using 1% agarose gels. Primers used are listed in Supplementary Table 1.

### Antibodies

Rat anti-mouse Vangl2 antibody was generated using Monoclonal Antibody Production facility (Luminy - Marseille, France). Vangl2 antibody was generated by immunizing rat with the GST-Vangl2 corresponding to amino-acids 1-102 (labeled as GST-NVangl2). Vangl2 monoclonal antibodies were screened using ELISA against the GST, GST-NVangl1 and GST-NVangl2 and in parallel with western blot. Antibodies from hybridoma supernatants were selected to specifically recognize Vangl2 protein only in tagged and untagged fusion proteins. Mouse anti-γ-tubulin (clone B-5-1-2) antibodies purchased from Sigma-Aldrich (France). Rabbit polyclonal anti-Vangl1/2 was previously described [21]. Anti-GFP polyclonal antibody (Abcam). Mouse anti-HA antibody and mouse anti-Myc (9E10) antibody (Santa Cruz). Secondary antibodies coupled to horseradish peroxidase were purchased from Dako (France).

### Immunoprecipitation

Cells were lysed with the lysis buffer consisting of 150 mM NaCl, 20 mM Hepes, 5 mM EDTA, 1% NP40 and supplemented with protease inhibitors (Sigma protease cocktail), phosphatase inhibitor cocktail (Sigma, phosphatase cocktail III) and orthovanadate. Immunoprecipitations were carried out as previously described [30]. Western blot analysis of cochlea taken from mice of different genetic backgrounds as previously described [21].

### Immunofluorescence

Cells were cultured on glass coverslips and fixed with 4% paraformaldehyde (PFA) for 15 min, permeabilised with 0.5% Triton x100 for 5 min and blocked with 10% fetal calf serum in PBS for 30 min before addition of phalloidin. Images were acquired using a Zeiss Axiovert 200 mot microscope linked to a Meta LSM 510 confocal module operated by LSM-FCS software (Carl Zeiss MicroImaging Inc, Germany) using 63X oil-immersion objective (Plan Achromatic, NA 1.3). Confocal image analyses were performed using LSM5 Image Browser software.

### Cochlea immunostaining

Cochleae from mice were dissected at specific time points and fixed in 4% PFA for 24 hr at 4°C. Cochleae whole mounts were processed as described previously (36) using anti-2G4 mAb (1∶100) and anti-Vangl1 (Sigma, 1/200) antibodies. Antibody binding was detected by using an Alexa Fluor-546- or -488 conjugated secondary antibodies (Invitrogen) at a dilution of 1∶300. When comparing staining between WT and mutants, both cochleae were processed in the same tube. Cell boundaries were visualized with phalloidin conjugated to Alexa-647 (Molecular Probes). After immunostaining, optical sectioning was obtained on a confocal microscope (TCS SP2; Leica). Imaging was done using a Z step from 0.3 to 0.4 µm. Confocal images were processed in Volocity software (Perkin Elmer) and Adobe Photoshop. Looptail (Lp) mutant mice on the LPT/Le stock were obtained from The Jackson Laboratory (Bar Harbor, ME). The mice were maintained at Magendie Neurocenter under standard conditions by heterozygous intercross. To generate homozygous mutants, heterozygous animals were crossed and timed-pregnant litters were obtained. [21]

### SPR analysis

SPR experiments in Figure 1D were performed at 25°C on a Biacore 3000 instrument (GE Healthcare) using HBS buffer (150 mM NaCl, 3 mM EDTA, 0.005% surfactant P20, 10 mM HEPES-NaOH, pH 7.4) as the running buffer. Polyclonal goat anti-GST antibodies (GE Healthcare) were covalently immobilized on sensor chips CM5 (GE Healthcare) using an amine coupling chemistry according to the manufacturer's instructions. Approximately 12–18 fmol of GST fusion proteins or GST were immobilized in experimental and control cells, respectively, *via* anti-GST antibodies. Monoclonal anti-Vangl2 antibody 2G4 (10 µg/ml) was injected at a flow rate of 25 µl/min. Non-specific adsorption to GST (control flow cell) generates less than 1% of the total signal. To measure the constant of dissociation, HBS buffer was supplemented with BSA 0.1%. Monoclonal anti-Vangl2 antibody 2G4 was covalently immobilized (6–6.5 fmol) on sensor chips CM5 (GE Healthcare) using an amine coupling chemistry according to the manufacturer's instructions. Irrelevant monoclonal antibody was coupled to the control flow cell as negative control. GST-NVangl2 was serially diluted 2-fold in running buffer yielding concentrations ranging from 4 to 66 nM and samples injected (90 s association time) over specific and control flow cells. Non-specific adsorption to irrelevant monoclonal antibody generates less than 3% of the total signal. Blank run injections of running buffer were performed in the same conditions. Substracted sensorgrams were globally fitted with the 1∶1 titration kinetic binding model from BiaEvaluation 4.1 software. Data are representative of three independent experiments.

SPR experiments in Figure S2 were performed at 25°C on a Biacore 3000 instrument (GE Healthcare) using HBS buffer (150 mM NaCl, 3 mM EDTA, 0.005% surfactant P20, BSA 0.1%, 10 mM HEPES-NaOH, pH 7.4) as the running buffer. Monoclonal anti-Vangl2 antibody was covalently immobilized (6–6.5 fmol) on sensor chips CM5 (GE Healthcare) using an amine coupling chemistry according to the manufacturer's instructions. Irrelevant monoclonal antibody was coupled to the control flow cell as negative control. GST-NVangl2 was serially diluted 2-fold in running buffer yielding concentrations ranging from 4 to 66 nM and samples injected (90 s association time) over specific and control flow cells. Non-specific adsorption to irrelevant monoclonal antibody generates less than 3% of the total signal. Blank run injections of running buffer were performed in the same conditions. The black trace represents the specific binding obtained after subtraction of a blank run and the red trace represents the fit of the data with 1∶1 titration kinetic binding model from BiaEvaluation 4.1 software. These data are representative of three independent experiments.

### Mass Spectrometry Analyses

The gel was initially silver stained and bands of the appropriate MW from Coomassie-stained gels were excised, digested with trypsin (Promega, Madison, WI, USA) and subjected to mass spectrometric analysis for identification. Proteins were identified by nanoLC-MSMS mass spectrometry analysis using an LTQ Orbitrap Velos mass spectrometer.

### Mass spectrometry analyses

Five microliters of peptide sample corresponding to 1/10 of extract was injected onto a nanoliquid chromatography system (nanoLC Ultimate 3000; Dionex, Sunnyvale, CA). After pre-concentration and washing of the sample on a Dionex Acclaim PepMap 100 C18 column (2 cm×100 µm i.d. 100 A, 5 µm particle size), peptides were separated on a Dionex Acclaim PepMap RSLC C18 column (15 cm×75 µm i.d., 100 A, 2 µm particle size) (Dionex, Amsterdam) using a linear 90 min gradient (4–40% acetonitrile/H_2_0; 0.1% formic acid) at a flow rate of 300 nL/min. Separation of the peptides was monitored by a UV detector (absorption at 214 nm). The nanoLC was coupled to a nanospray source of a linear ion trap Orbitrap mass spectrometer (LTQ Orbitrap Velos, Thermo Electron, Bremen, Germany). The LTQ spray voltage was 1.4 kV and the capillary temperature was set at 275°C. All samples were measured in a data dependent acquisition mode. Each run was preceded by a blank MS run in order to monitor system background. The peptide masses are measured in a survey full scan (scan range 300–1700 m/z, with 30 K FWHM resolution at m/z = 400, target AGC value of 1.00×106 and maximum injection time of 500 ms). In parallel to the high-resolution full scan in the Orbitrap, the data-dependent CID scans of the 10 most intense precursor ions were fragmented and measured in the linear ion trap (normalized collision energy of 35%, activation time of 10 ms, target AGC value of 1.00×104, maximum injection time 100 ms, isolation window 2 Da). The fragment ion masses are measured in the linear ion trap to have a maximum sensitivity and the maximum amount of MS/MS data. Dynamic exclusion was implemented with a repeat count of 1 and exclusion duration of 37 s

### Data Analysis

Raw files generated from mass spectrometry analysis were processed with Proteome Discoverer 1.1 (Thermo fisher Scientific). This software was used to search data via in-house Mascot server (version 2.2.03; Matrix Science Inc., London, UK) against the Human database subset of the Uniprot database. For the database search the following settings were used: a maximum of one miscleavage, oxidation as a variable modification of methionine, carbamido-methylation as a fixed modification of cysteine and trypsin was set as the enzyme. A peptide mass tolerance of 5 ppm and a fragment mass tolerance of 0.8 Da were allowed. An ion score of 30 was used as a cut-off value.

### Western Blotting

Proteins were separated using SDS-PAGE and transferred to nitrocellulose membranes. Non-specific antibody binding was blocked with 5% BSA, 1×Tris buffered saline (TBS) (10×TBS (0.5 M Tris-Cl, 150 mM NaCl, pH 7.6), 1% Tween-20® and membranes were incubated with primary antibodies for 2 hours at room temperature. Species-specific HRP conjugated secondary antibodies were used for detection of primary antibodies and developed with a chemioluminescent substrate (Pierce).

## Supporting Information

Figure S1 Raw ELISA data for all Vangl2 antibody clones tested with the antibody clones in Figure 1B highlighted with an asterisk.
(TIF)

Figure S2 SPR experiment and calculation of K_D_, K_off_ and K_on_ constants of association for Vangl2G4 antibody with GST-NVangl2 antigen.
(TIF)

Figure S3 Immunoreactivity of the amino acids 1-20 of N terminal Vangl2 fused with GST protein and detected using Vangl2G4 antibody, with appropriate Coomassie-stained SDS gel and GST western blot of purified proteins.
(TIF)

Figure S4 Expression of Vangl1 and Vangl2 in breast cancer cells. (**A**) Expression of Vangl1 and Vangl2 mRNA in different breast cell lines assessed using total-RNA, retro-transcribed to cDNA and used in PCR reaction with GADPH controls. (**B**) Protein expression of endogenous Vangl1 and Vangl2 in breast cancer cell lines detected with Vangl1/2 antibody or 2G4 mAb.
(TIF)

Figure S5 Immunoprecipitation products (SKRB7 cell lysates) separated using a 16% SDS-PAGE
(TIF)

Figure S6 Spectra corresponding to a peptide appearing in the extended N-terminal of Vangl2.
(TIF)

Figure S7 2G4 mAb immunoprecipitations using SKBR7 cells transfected with shLuc or shVangl2.
(TIF)

Figure S8 Transient co-expression of myc-Vangl1 with GFP-Vangl2 in T47D cells, and immunoprecipitation with 2G4 mAb shows co-immunoprecipitation of Vangl1 with Vangl2.
(TIF)

Figure S9 (A) Specificity of the 2G4 mAb in western blot was shown using protein extracts of SUM149 cells transfected with shLuc or shVangl2. (B) Similar experiment as (A) using SKBR7 cells treated with 2 different siRNA against Vangl2 or with control siRNA (non-targeting siRNA).
(TIF)

Table S1 List of primers used in RT-PCR experiments for the screening of mRNA expression levels of Vangl1 and Vangl2.
(DOC)
