# Supplementary figures and images for: Molecular Characterisation of Endogenous Vangl2/Vangl1 Heteromeric Protein Complexes
Source: PLoS One. 2012 Sep 28;7(9):e46213. doi: 10.1371/journal.pone.0046213 (PMC3460870; doi:10.1371/journal.pone.0046213)

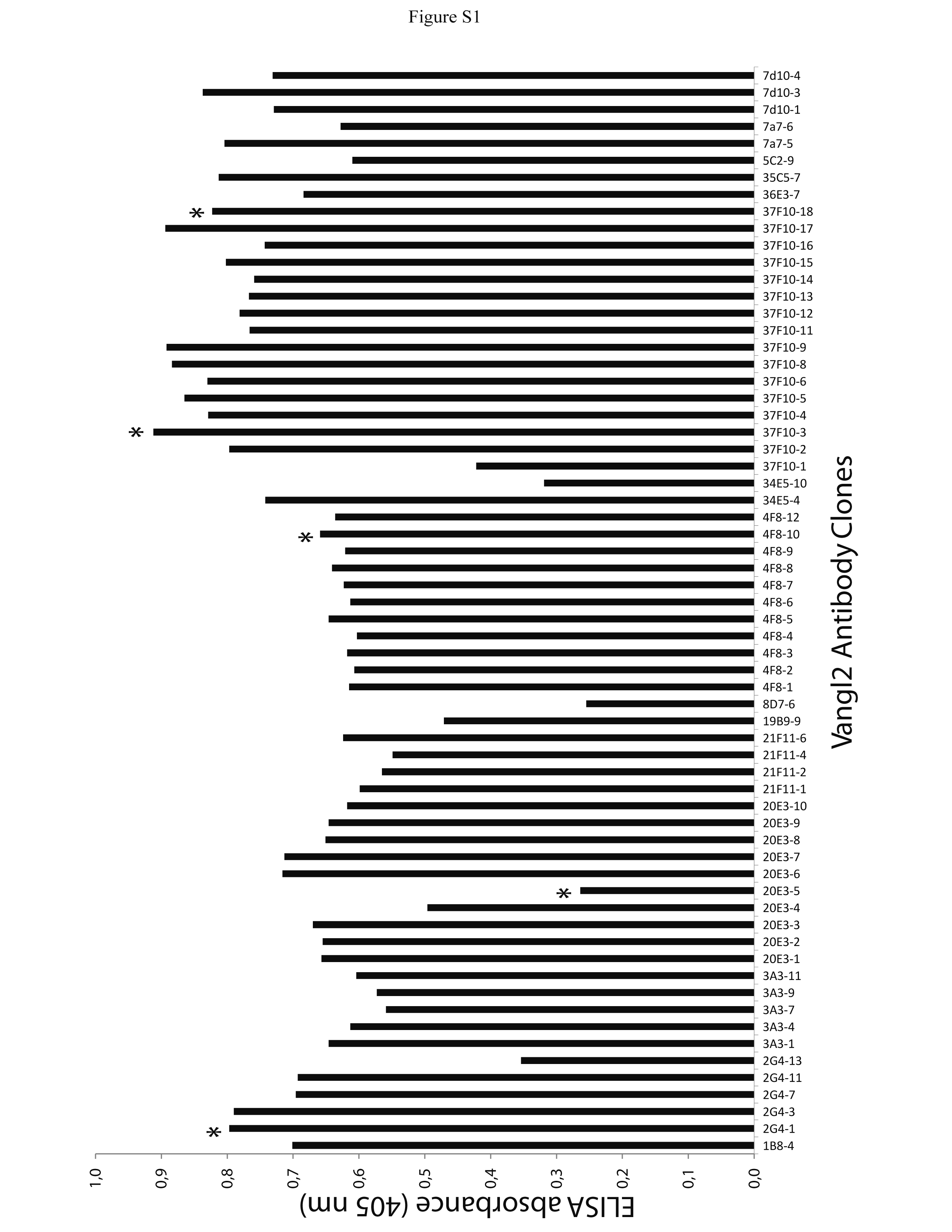

Supplement: Figure S1 — Raw ELISA data for all Vangl2 antibody clones tested with the antibody clones in Figure 1B highlighted with an asterisk. (TIF) [file pone.0046213.s001.tif]

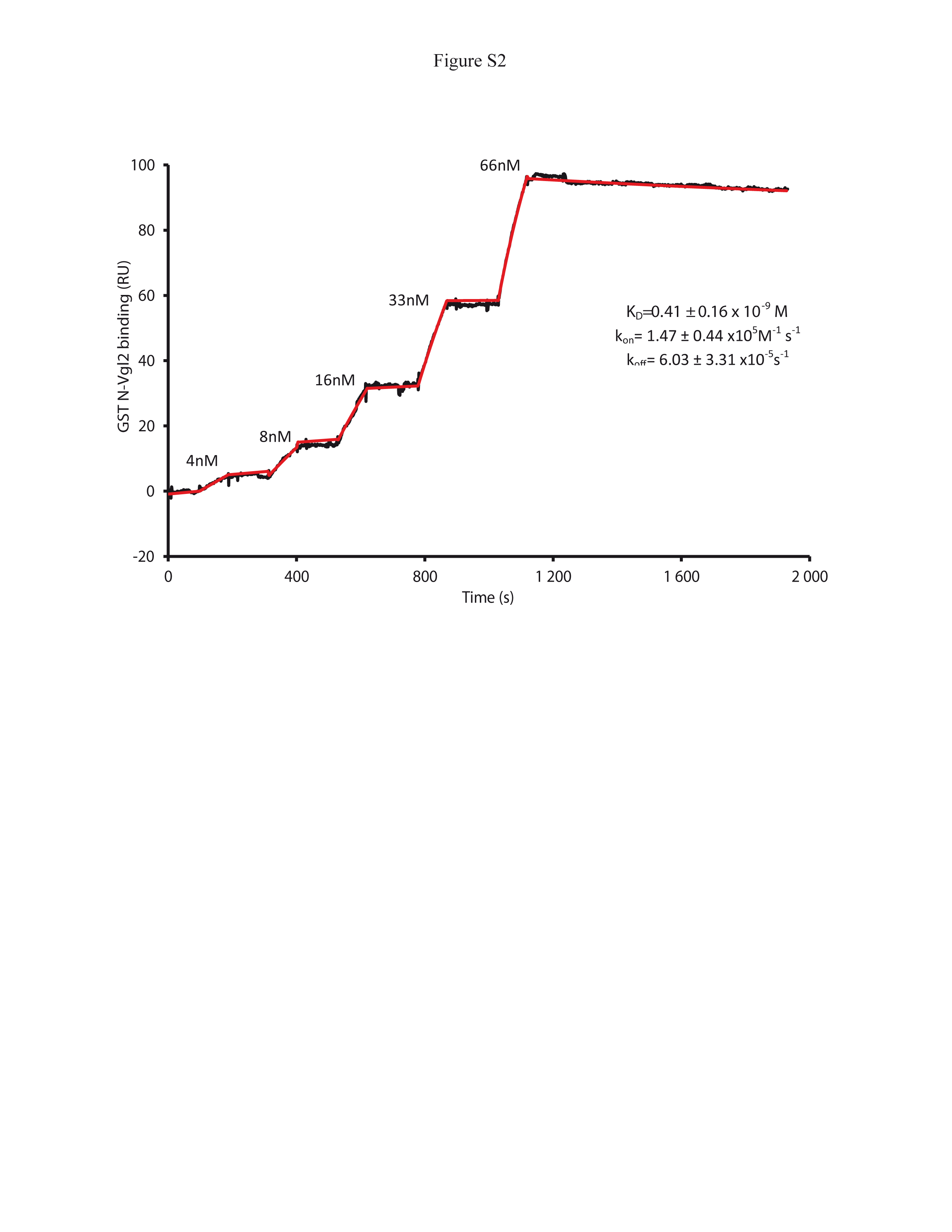

Supplement: Figure S2 — SPR experiment and calculation of KD, Koff and Kon constants of association for Vangl2G4 antibody with GST-NVangl2 antigen. (TIF) [file pone.0046213.s002.tif]

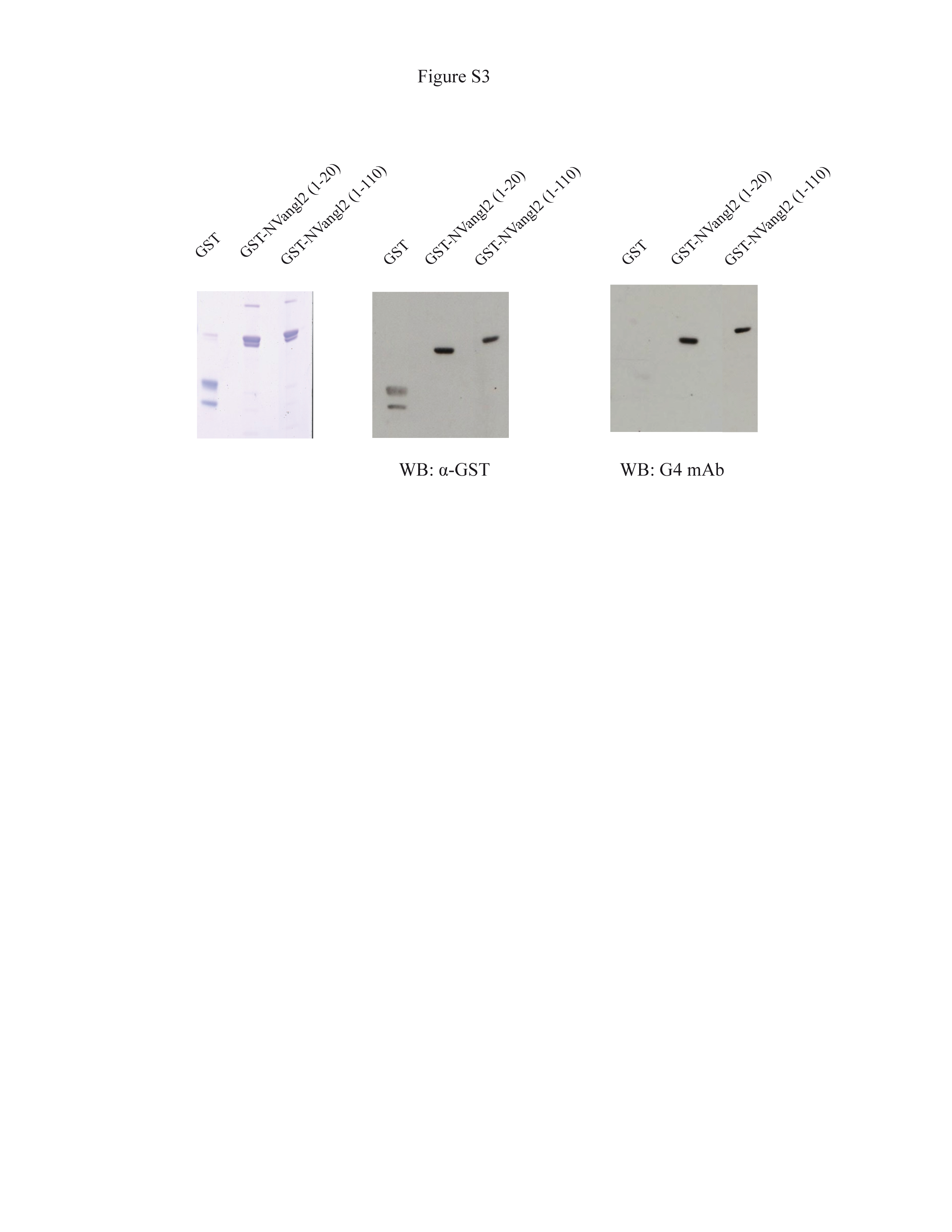

Supplement: Figure S3 — Immunoreactivity of the amino acids 1-20 of N terminal Vangl2 fused with GST protein and detected using Vangl2G4 antibody, with appropriate Coomassie-stained SDS gel and GST western blot of purified proteins. (TIF) [file pone.0046213.s003.tif]

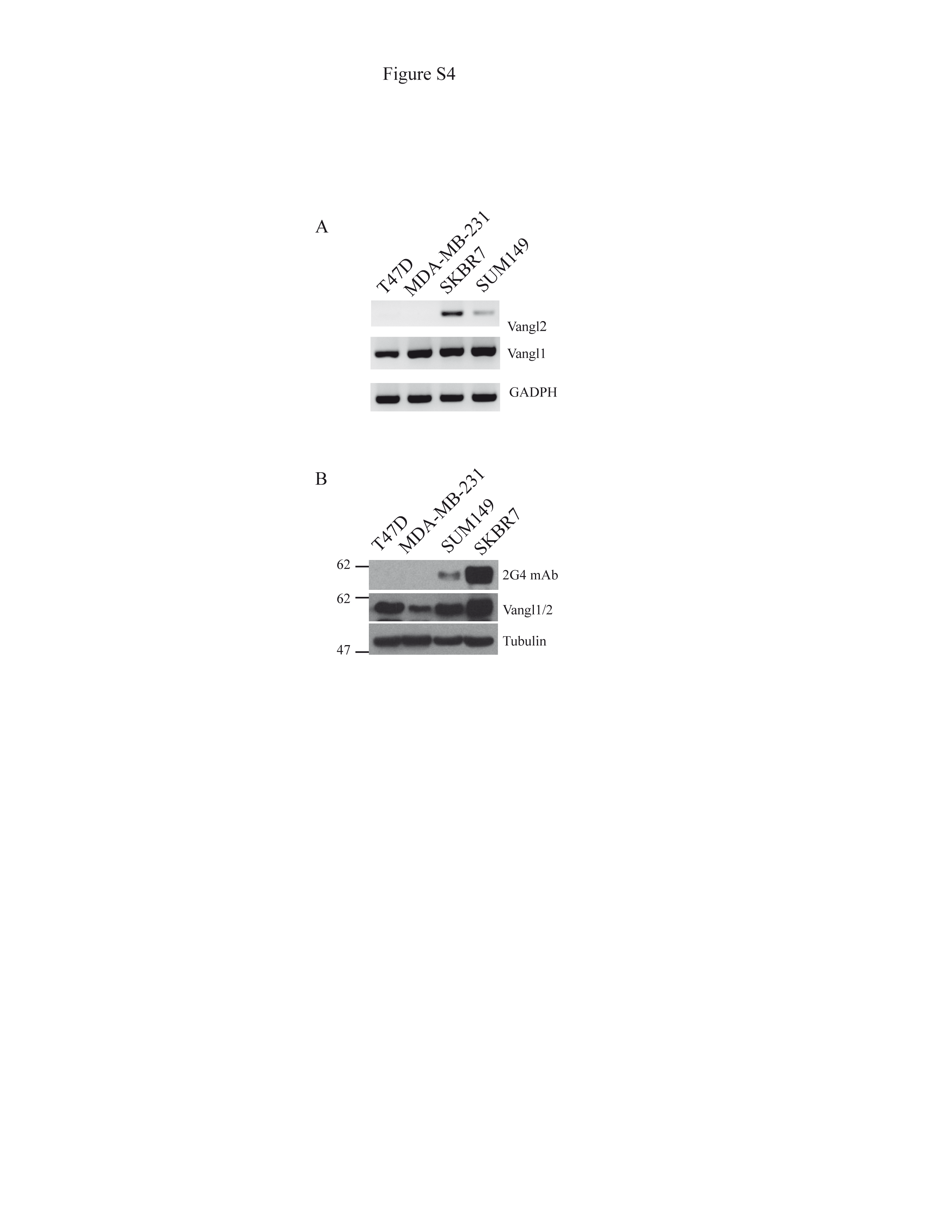

Supplement: Figure S4 — Expression of Vangl1 and Vangl2 in breast cancer cells. (A) Expression of Vangl1 and Vangl2 mRNA in different breast cell lines assessed using total-RNA, retro-transcribed to cDNA and used in PCR reaction with GADPH controls. (B) Protein expression of endogenous Vangl1 and Vangl2 in breast cancer cell lines detected with Vangl1/2 antibody or 2G4 mAb. (TIF) [file pone.0046213.s004.tif]

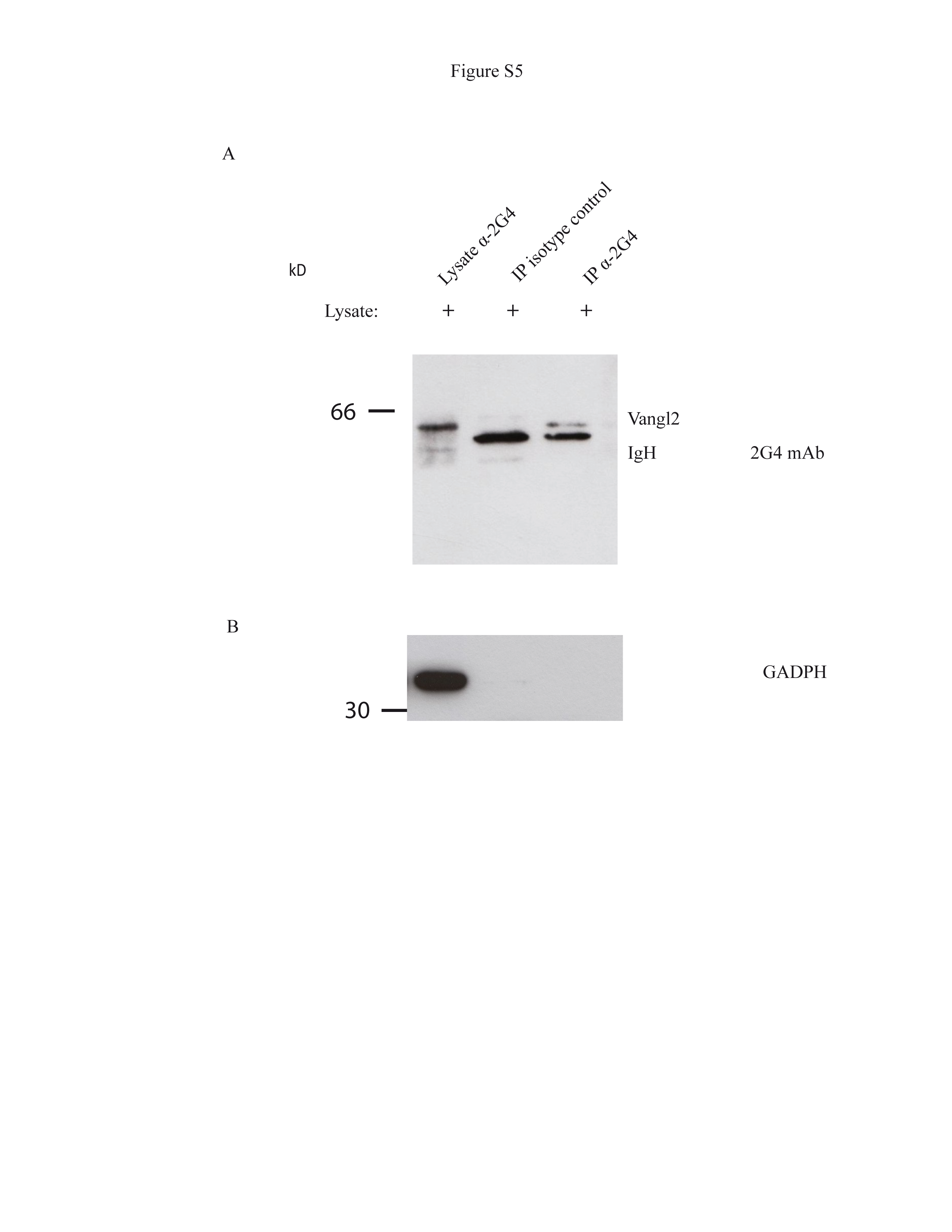

Supplement: Figure S5 — Immunoprecipitation products (SKRB7 cell lysates) separated using a 16% SDS-PAGE (TIF) [file pone.0046213.s005.tif]

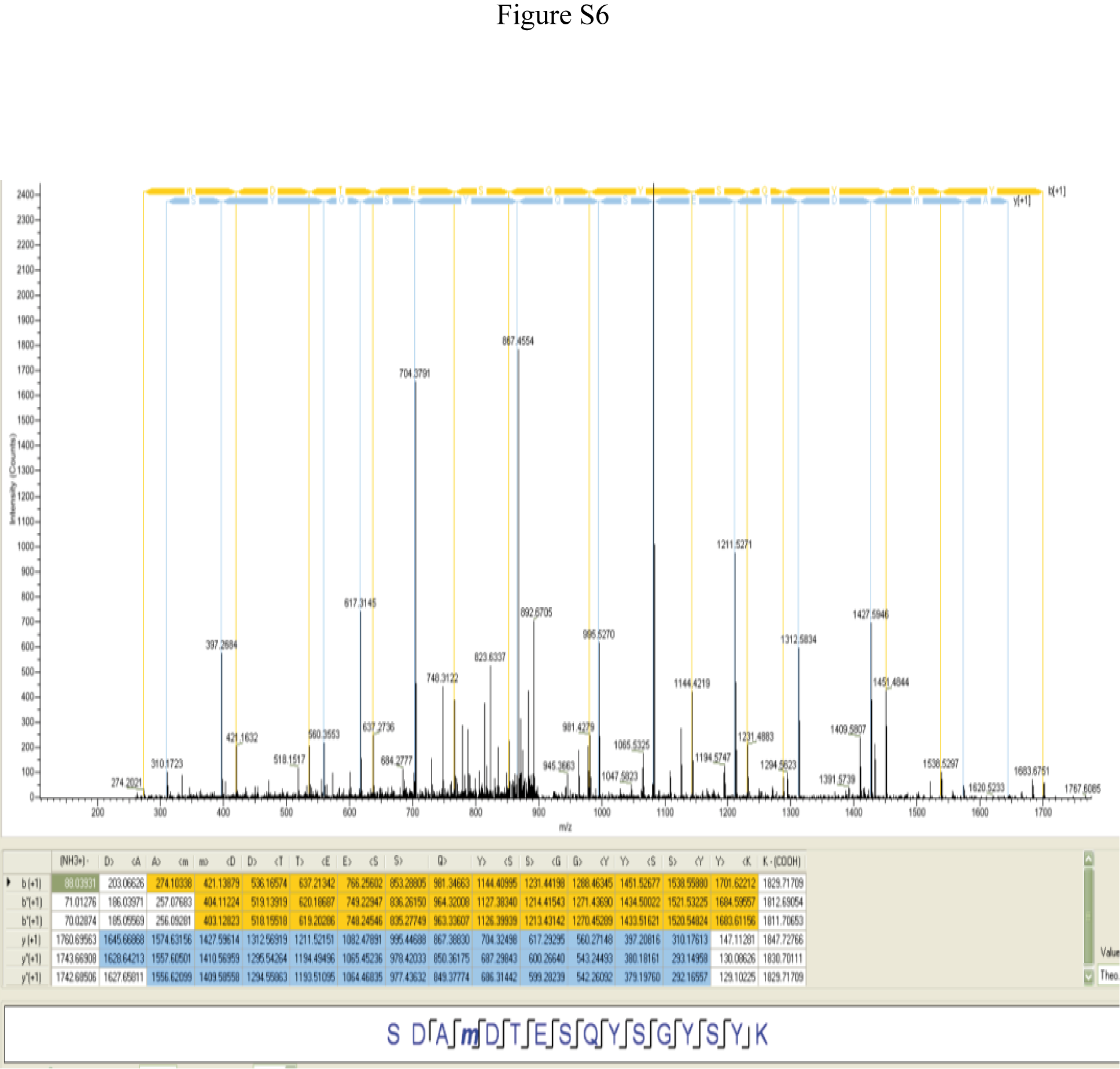

Supplement: Figure S6 — Spectra corresponding to a peptide appearing in the extended N-terminal of Vangl2. (TIF) [file pone.0046213.s006.tif]

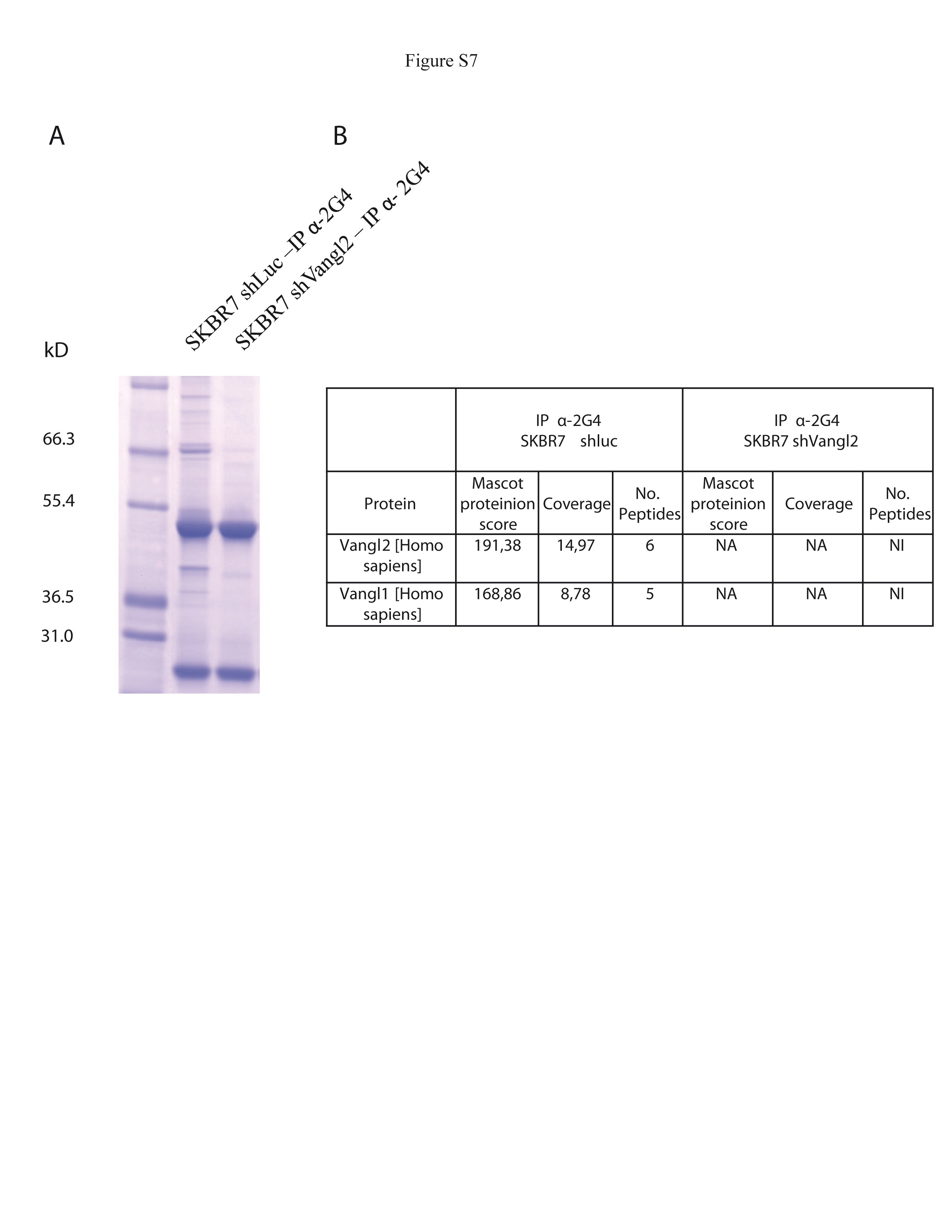

Supplement: Figure S7 — 2G4 mAb immunoprecipitations using SKBR7 cells transfected with shLuc or shVangl2. (TIF) [file pone.0046213.s007.tif]

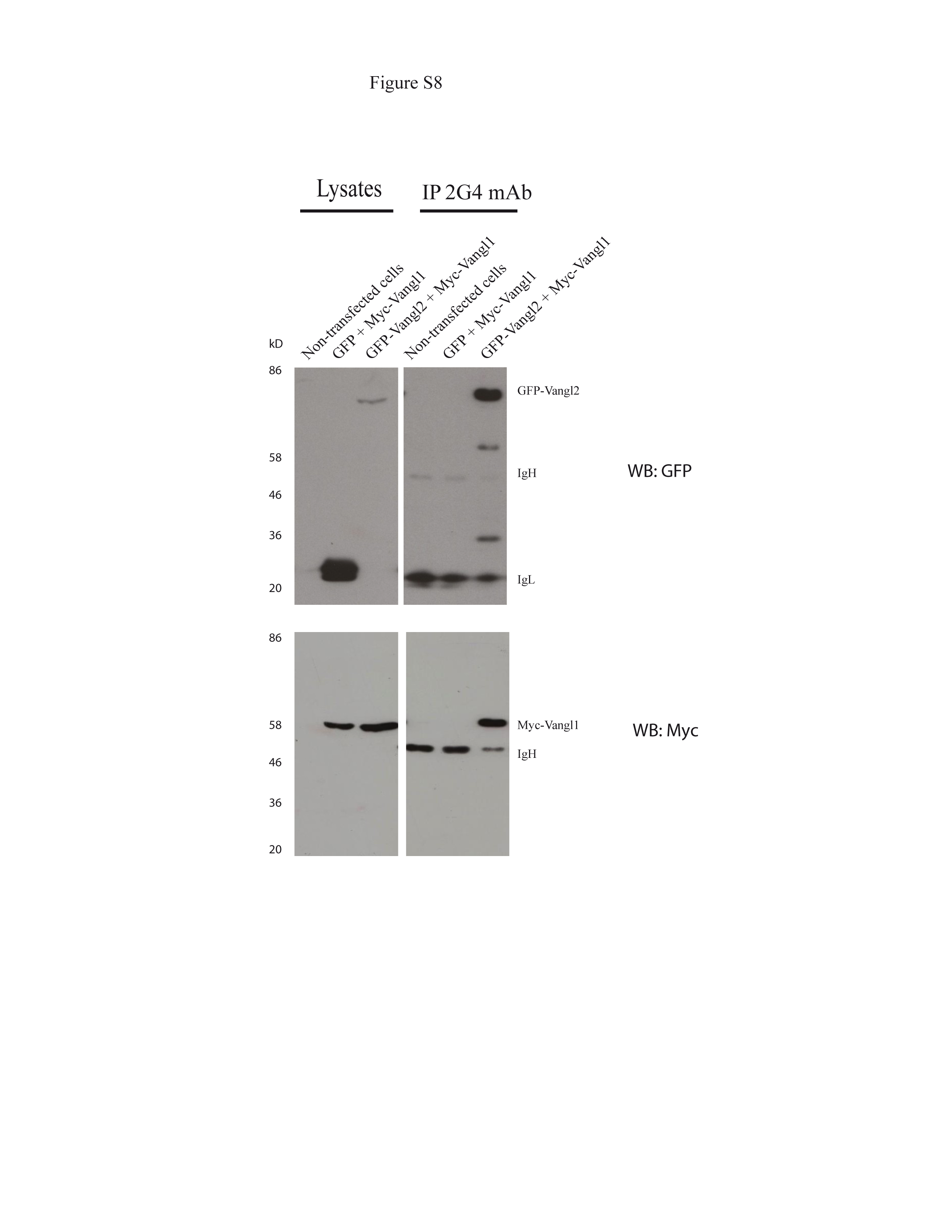

Supplement: Figure S8 — Transient co-expression of myc-Vangl1 with GFP-Vangl2 in T47D cells, and immunoprecipitation with 2G4 mAb shows co-immunoprecipitation of Vangl1 with Vangl2. (TIF) [file pone.0046213.s008.tif]

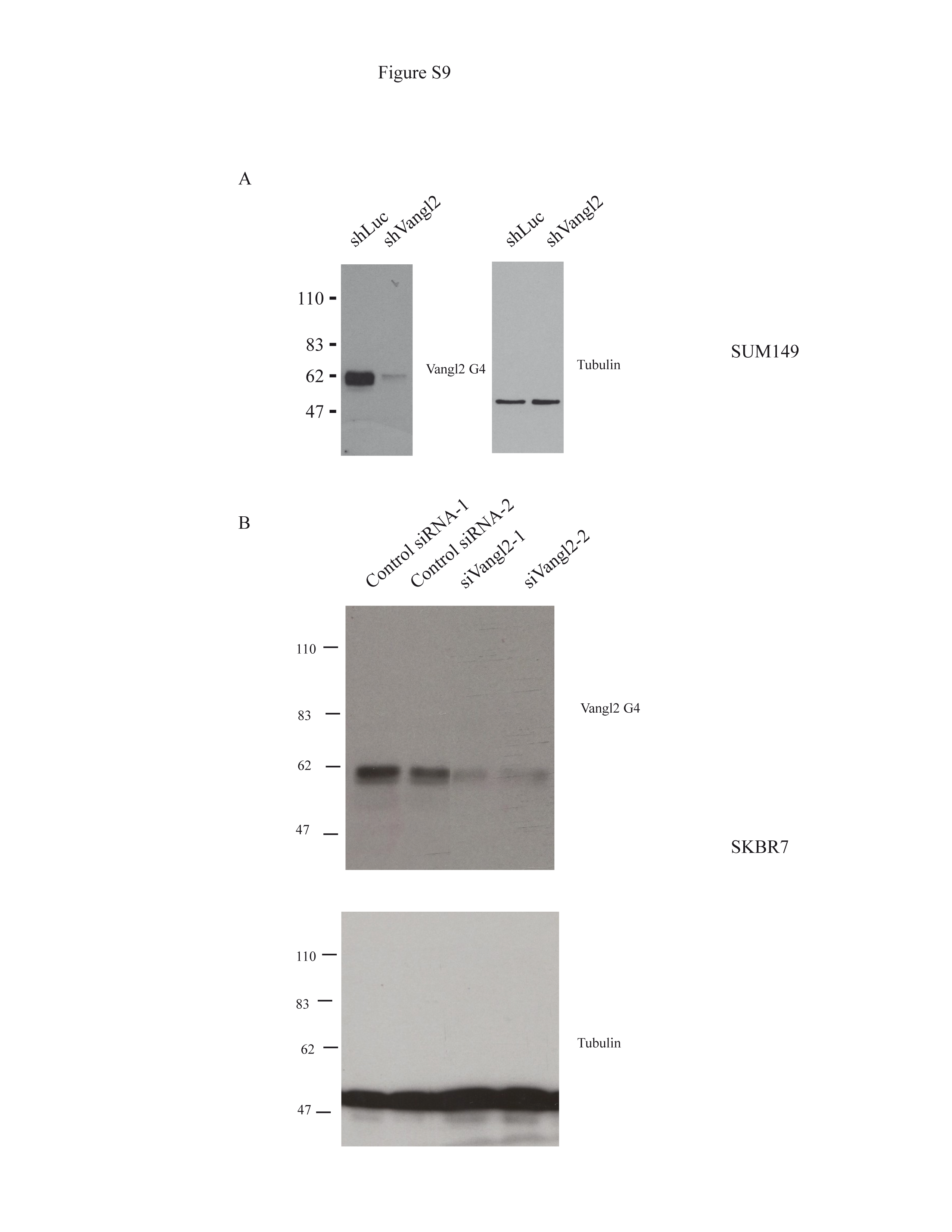

Supplement: Figure S9 — (A) Specificity of the 2G4 mAb in western blot was shown using protein extracts of SUM149 cells transfected with shLuc or shVangl2. (B) Similar experiment as (A) using SKBR7 cells treated with 2 different siRNA against Vangl2 or with control siRNA (non-targeting siRNA). (TIF) [file pone.0046213.s009.tif]
