# Supplementary material for: Molecular Characterisation of Endogenous Vangl2/Vangl1 Heteromeric Protein Complexes
Source: PLoS One. 2012 Sep 28;7(9):e46213. doi: 10.1371/journal.pone.0046213 (PMC3460870; doi:10.1371/journal.pone.0046213)
Supplement: Table S1 — List of primers used in RT-PCR experiments for the screening of mRNA expression levels of Vangl1 and Vangl2. (DOC) [file pone.0046213.s010.doc]

**Table S1-** List of primers used in RT-PCR experiments for the screening of mRNA expression levels of Vangl1 and Vangl2.

| **Gene** |  |
| --- | --- |
| GAPDH forward | 5’-CCACCCATGGCAAATTCCATGGCA-3’ |
| GADPH reverse | 5’-GGTGGACCTGACCTGCCGTCTAGA-3’(200 bp product) |
| VANGL1 forward | 5’-CTTCAGCCTCGTAGTCAATGTG-3’ |
| VANGL1 reverse | 5’-CCTTTCGTCCAGTACCAAGTCC-3’ (434 bp product) |
| VANGL2 forward | 5’-TGAATTCTGCATCACGCATGAC-3’ |
| VANGL2 reverse | 5’-GCACCAAGAAGGTCCCATTCTT-3’ (220 bp product) |
